# Influence of Fe Ions on Anode Performance and the Mechanism of Action during Copper Electrowinning Process

**DOI:** 10.3390/molecules29194578

**Published:** 2024-09-26

**Authors:** Cheng Jiang, Yiwen Chen, Yingping Zhou, Buming Chen, Hui Huang, Jun Guo, Ruidong Xu, Zhongcheng Guo

**Affiliations:** 1 Faculty of Metallurgical and Energy Engineering, Kunming University of Science and Technology, Kunming 650093, China; jiangcheng20220419@163.com (C.J.);; 2 State Key Laboratory of Comprehensive Utilization of Low-Grade Refractory Gold Ores, Xiamen 361101, China; 3 Research Center of Metallurgical Electrode Materials Engineering Technology, Kunming 650106, China; 4 Kunming Hendera Science and Technology Co., Ltd., Kunming 650106, China

**Keywords:** surface reaction, oxygen evolution reaction, corrosion resistance, Fe doping

## Abstract

The performance of the anode varies from the impurity ions in the copper electrowinning process. This work focused on the variation of the electrochemical behavior of the Pb-0.06%Ca-1.2%Sn anode as the Fe ions (Fe^3+^ and/or Fe^2+^) existed in the electrolyte by electrochemical characterization. Copper electrodeposition experiments were conducted under a current density of 300 A/m^2^, with the Fe ion concentration in the electrolyte controlled within the range of 0 to 20 g/L and the Cu ion concentration maintained at 45 g/L at a temperature of 45 °C. The variation in the corrosion resistance, catalytic activity, and structural composition of the anode film layer was analyzed in-depth according to the presence of Fe ions. The results show that the structure of PbO_2_ on the surface of the film was changed as Fe ions doped into the anode film, and the oxygen evolution activity of the anode was also improved. However, the corrosion resistance decreased with increasing Fe^3+^ concentration. Furthermore, the addition of 2 g/L Fe^2+^ in the electrolyte containing 2 g/L Fe^3+^ led to an elevation in the corrosion resistance of the anode to some extent and further increased the oxygen evolution activity.

## 1. Introduction

Copper metal, renowned for its exceptional electrical and thermal conductivity alongside other metallic properties, finds widespread application in everyday necessities such as electricity, electronics, construction, transportation, industrial machinery manufacturing, and consumer goods [1]. Approximately 20% of industrial copper is obtained through electrolytic deposition, employing Pb−Ca−Sn alloy anodes in the electrodeposition process [2]. While much attention has been directed towards understanding the impact of impurity ions on cathode copper in the electrodeposition process, such as Co^2+^ [3,4], Ge^3+^ [5], Ni^2+^ [6,7], and Sb^3+^ [8], it is imperative to recognize that impurity ions also significantly influence the behavior of the anode.

During copper electrolysis, impurity ions like Mn [9] lead to unforeseen corrosive effects and other alterations on the anode, whereas Co ions exhibit favorable effects on lead anodes [10]. As the MnO_2_ layer forms and its thickness increases, the conductivity of the membrane decreases, and the oxygen evolution activity declines. The thickness of the MnO_2_ layer increases with higher manganese concentrations in the solution. However, the protective effect of the MnO_2_ layer does not improve linearly with increasing manganese ion concentration. This is attributed to the presence of an inert PbSO_4_ layer between the MnO_2_ and PbO_2_ layers. Consequently, as the MnO_2_ layer thickness increases, MnO_2_ becomes more prone to detachment, leading to the dissolution of the anode lead [11,12]. By adding Co^2+^ to the solution, the overpotential for the oxygen evolution reaction (OER) on the Pb anode decreases, and the potential stabilizes rapidly during constant current polarization. Additionally, the corrosion resistance of the Pb anode is significantly improved. This is primarily due to the oxidation of Co^2+^ to Co^3+^ during the OER process. Co^3+^ exhibits excellent catalytic activity for the OER, thereby enhancing the overall OER performance [13]. Additionally, there are documented effects of other metal ions, such as Ag^+^ and Al^3+^ [14,15,16]. In addition to the above impurity ions, Fe ions also play an important role in the hydrometallurgical copper refining process. Fe ions usually enter the electrolyte with the extraction process and exist in the electrolyte in the form of Fe^3+^ [17]. As the Fe content increases, the current efficiency gradually decreases. This phenomenon is attributed to the complex behavior of iron ions in the electrolyte, where Fe^2^⁺ is oxidized to Fe³⁺ at the anode and Fe³⁺ is reduced back to Fe^2^⁺ at the cathode [18]. This conversion occurring at the cathode affects copper deposition and reduces current efficiency. However, the presence of Fe can enhance the catalytic activity of the anode, effectively improving its catalytic performance and reducing energy consumption [17,19,20]. Current research has not yet investigated the role of the anode in the copper electrolysis process. Therefore, studying the impact of iron ions on the anode during the copper electrolysis process and understanding its mechanisms is important.

This study aims to investigate the impact of iron ions in the electrolyte on the anode during copper electrolysis. The electrochemical behavior of a Pb−0.06%Ca−1.2%Sn anode in iron-ion-containing electrolytes was examined, and the performance of the lead alloy anode was evaluated. In addition, a detailed analysis of the surface morphology and phase structure of the anode film, along with corresponding electrochemical results, was conducted to explore the role of iron ions. Specifically, the mechanisms underlying changes in the anode film in iron-ion-containing electrolytes were elucidated, confirming the effect of Fe ion concentration on the anode and current efficiency during copper electrodeposition. The optimal concentration of Fe ions in the copper electrolyte was identified, demonstrating that controlling the Fe concentration in the electrolyte during electrodeposition can enhance the oxygen evolution reaction catalytic activity of the anode, reduce energy consumption, and provide a theoretical foundation for the development of copper electrolysis in hydrometallurgical industries.

## 2. Experiment Section

### 2.1. Electrolyte Composition

The experiment utilized an electrolyte containing 180 g/L H_2_SO_4_ and 45 g/L Cu^2+^. To investigate the effects of different iron ion concentrations on the anode, the Fe^2+^ content in the electrolyte was controlled by adjusting the amount of FeSO_4_ added, while the Fe^3+^ content was controlled by varying the amount of Fe_2_(SO_4_)_3_ added, maintaining Fe^2+^ ion concentrations at 0 g/L, 2 g/L, 4 g/L, 8 g/L, and 16 g/L and Fe^3+^ ion concentrations at 0 g/L, 2 g/L, 4 g/L, 8 g/L, and 16 g/L. When exploring the effect of Fe^3+^ concentration, only Fe_2_(SO_4_)_3_ was added to adjust Fe^3+^ levels. For studying the combined effects of Fe^2+^ and Fe^3+^, the Fe^3+^: Fe^2+^ concentration ratios were adjusted to 1:1, 1:2, 1:4, and 1:8. Analytical-grade reagents were used throughout the experiment to prevent interference from other ions in the electrolyte composition.

### 2.2. Anode Specimen Preparation and Electrochemical Testing

The anode specimens utilized in the experiments were fabricated as Pb−0.06%Ca−1.2%Sn anodes, where the percentage represents the mass fraction, while a stainless steel cathode was employed. Electrochemical specimens measuring 10 mm × 10 mm × 6 mm were prepared for the anodes, and they underwent polishing on a metallographic sample-making machine using sandpaper to ensure a test area of 10 mm × 10 mm and a smooth surface. The prepared anodes were then subjected to copper electrowinning experiments in electrolytes with varying Fe ion concentrations at a current density of 300 A/m^2^ for 48 h to observe the effect of Fe ions on the anode film layer.

The electrochemical tests were conducted using a three-electrode system, where the reference electrode was the saturated potassium sulfate electrode Hg/Hg_2_SO_4_/K_2_SO_4_ (0.645 V/MSE), the counter electrode was the platinum electrode, and the working electrode was the anode for completing the 48 h copper electrowinning experiments. All potentials in the experiments were referenced to the MSE reference electrode, and the specimens were immersed in 180 g/L H_2_SO_4_, 45 g/L Cu^2+^ solutions containing varying concentrations of Fe^3+^ ions, then electrochemically tested at a temperature of 45 °C. Each test was replicated three times to ensure stable and reliable experimental results. The error range for the three electrochemical experiments fell between 10% and 20%. However, the experimental results consistently displayed the same trend, indicating that the obtained electrochemical data were generally reliable. All electrochemical data presented in the manuscript carried an approximate error margin of 10%. Initial constant current polarization was conducted at 300 A/m^2^ for 30 min to ascertain the impact of impurity ions on the anode film layer. The catalytic oxygen evolution performance of the film layer was investigated through electrochemical impedance spectroscopy (EIS) at the oxygen evolution potential of 1.837 V (vs. RHE). The frequency range for the scan was from 100 kHz to 1 Hz, with an AC amplitude perturbation of 5 mV. The corrosion resistance of the electrode was evaluated using potentiodynamic polarization curve testing. The scan range was set to ±400 mV relative to the open-circuit potential, with a scan rate of 5 mV/s. Finally, anodic polarization curves (LSV) and cyclic voltammetry (CV) tests were conducted from the initial potential of 0.7 V to the final potential of 2.5 V (vs. RHE) at a constant scanning rate of 5 mV/s to investigate the anodic oxygen evolution reaction and the changes in the redox process influenced by varying Fe ion concentrations.

### 2.3. Physical Characterization

Following the completion of the experiments, the surface morphology of the specimens was examined using a scanning electron microscope (SEM) (VEGA4, TESCAN, Brno, Czech Republic) to explore alterations in the oxide film morphology on the anode surface influenced by varying Fe ion concentrations. Additionally, the elemental composition of different phases was analyzed using energy-dispersive spectroscopy (EDS) (VEGA4, TESCAN, Brno, Czech Republic). The phase composition of various samples was determined through XRD analysis (D2 Phaser, Bruker, Mannheim, Germany) to investigate the impact of differing Fe ion concentrations on phase changes. XPS analysis (NexsaXPS, Thermofisher Scientific, Waltham, MA, USA) was conducted to assess changes in ionic valence within the film layer, elucidating the reaction mechanism of Fe ions during copper electrowinning and clarifying modifications in the surface film layer.

## 3. Results and Discussion

### 3.1. Changes in Electrochemical Behavior of Anode under the Effect of Fe^3+^

**Figure 1:**
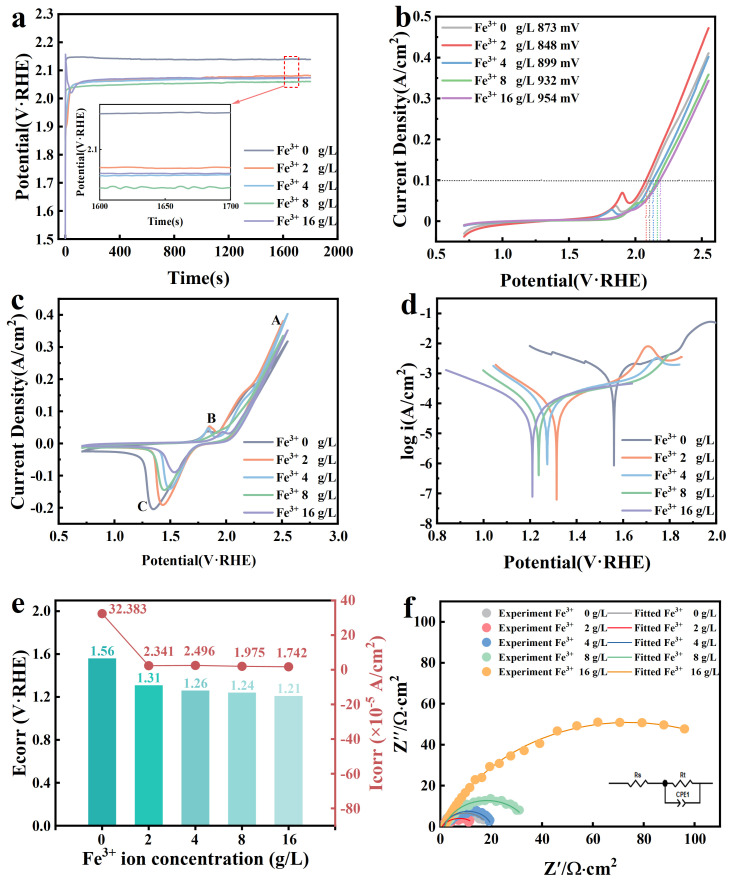
(**a**) Constant current polarization curves at different Fe^3+^ concentrations, (**b**) LSV curves at different Fe^3+^ concentrations, (**c**) CV curves at different Fe^3+^ concentrations, (**d**) Tafel curves at different Fe^3+^ concentrations, (**e**) anodic self−corrosion potentials and self−corrosion current densities, (**f**) EIS plots at different Fe^3+^ concentrations.

To investigate the effect of Fe^3+^ on the anodic performance during copper electrodeposition, experiments were conducted for 48 h in copper electrolyte solutions with varying Fe^3+^ concentrations. The experiments were conducted at a current density of 300 A/m^2^ and an electrolyte temperature of 45 °C to ensure the formation of a stable Pb−Ca−Sn anodic membrane. Electrochemical tests were conducted to examine the oxygen evolution activity, corrosion resistance, and phase changes of the anode, clarifying the effect of Fe^3+^ on the anode. The constant current polarization curves of the anode under different Fe^3+^ concentrations, as shown in Figure 1a, reflect the stability of the anode film layer formation when Fe^3+^ was added. With the addition of Fe^3+^, the anode potential rapidly decreased, showing a significant change compared to when Fe^3+^ was not present, indicating that Fe^3+^ reduced the reaction potential to some extent, thereby lowering energy consumption. When the Fe^3+^ concentration reached 16 g/L, a peak appeared in the curve at the beginning of the reaction, indicating that with increasing Fe^3+^ concentration, the initial polarization process at the anode became more complex. At this point, the surface membrane formed PbSO_4_ and PbO_2_, while Fe^3+^ from the electrolyte concurrently entered the anodic film in the form of Fe_2_O_3_.

The oxygen evolution reaction (OER) is well represented by the LSV shown in Figure 1b. By comparing the oxygen evolution overpotential (η_OER_) at a current density of 100 mA/cm^2^, it was observed that at a Fe^3+^ concentration of 2 g/L, the OER overpotential was around 848 mV, indicating good catalytic activity and reduced energy consumption. However, as the Fe^3+^ concentration continued to increase, the OER potential rose instead. This suggests that an excessive Fe^3+^ concentration alters the structure of the anode film layer, hindering the OER process and reducing the catalytic activity of the anode. The CV curves shown in Figure 1c further illustrate the impact of Fe^3+^ on the redox processes and catalytic activity of the anode [21]. Peak A in the oxidation curve shows changes in the oxygen evolution reaction. With the addition of Fe^3+^, the peak intensity initially increased and then decreased. When the Fe^3+^ concentration was 2 g/L, the peak intensity was highest, indicating that Fe^3+^ at 2 g/L enhanced the oxygen evolution catalytic activity at the anode in the electrolyte. Oxidation peak B shifted to the right, indicating that the potential required for the conversion of PbO_x_ (0 < x < 1) to PbSO_4_ increased with Fe^3+^. This resulted in higher energy consumption and a reduced formation of PbSO_4_. Additionally, the intensity of the reduction peak C decreased with the addition of Fe^3+^, suggesting that the conversion of PbO_2_ in the film layer was hindered, leading to a decrease in PbO_2_ formation. As the Fe^3+^ concentration rose, the content of PbSO_4_ and PbO_2_ in the anode film layer decreased, resulting in reduced corrosion resistance and catalytic activity of the anode.

The Tafel curves acquired from electrochemical tests serve as a reference for assessing anode corrosion resistance. From these curves, anodic self-corrosion potentials and corrosion current densities under varying Fe^3+^ concentrations are extrapolated [22]. As shown in Figure 1d, with increasing Fe^3+^ concentration, the Tafel curves shifted progressively to the left and downward. The corresponding anode self-corrosion potential in Figure 1e also decreased. This indicates that higher Fe^3+^ concentrations resulted in a lower potential required for anode corrosion, increasing the thermodynamic tendency for corrosion. This suggests that the reduction in PbSO_4_ and PbO_2_ content in the film layer made the anode more susceptible to corrosion. Figure 1f presents the Nyquist plot for analyzing the catalytic activity of the anode during the reaction. The charge transfer resistance (R_ct_) reflects the extent of hindrance to the oxygen evolution reaction (OER), with an increase in R_ct_ indicating a decrease in OER capability. At a Fe^3+^ concentration of 2 g/L, R_ct_ was lower compared to when no Fe^3+^ is present. As the Fe^3+^ concentration increased further, R_ct_ rose, indicating increased hindrance to the charge transfer process. This is attributed to the further reduction in PbO_2_ content in the anode film layer with higher Fe^3+^ concentrations, leading to a decline in anode catalytic activity. Table 1 shows that the roughness factor (R_F_) was related to the surface of the anode film layer, with R_F_ being highest at a Fe^3+^ concentration of 2 g/L. A higher R_F_ corresponds to a rougher anode surface, which increases the surface area available for the OER reaction and promotes its progress. This explains why the anode with 2 g/L Fe^3+^ exhibited better oxygen evolution activity despite the similar R_ct_ values [23,24].

### 3.2. Changes in the Electrochemical Behavior of the Anode in the Presence of Fe^2+^ and Fe^3+^

**Figure 2:**
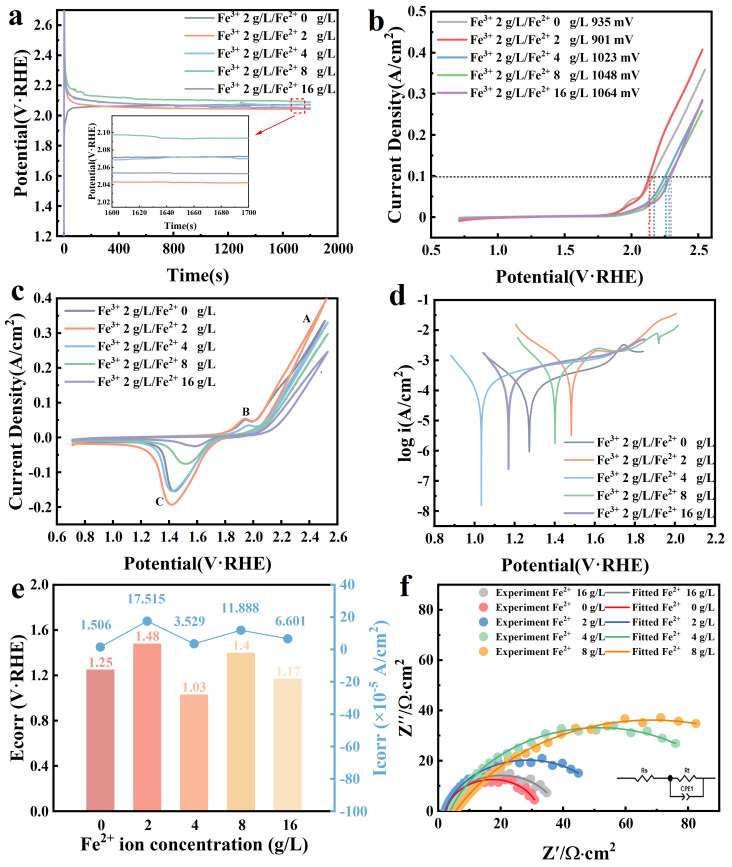
(**a**) Constant current polarization curves at different Fe^2+^ concentrations, (**b**) LSV curves at different Fe^2+^ concentrations, (**c**) CV curves at different Fe^2+^ concentrations, (**d**) Tafel curves at different Fe^2+^ concentrations, (**e**) anodic self−corrosion potentials and self−corrosion current densities, (**f**) EIS plots at different Fe^2+^ concentrations.

To further investigate the influence of coexisting Fe^2+^ and Fe^3+^ in the electrolyte on traditional lead anodes, an electrolyte with excellent oxygen evolution activity at a Fe^3+^ concentration of 2 g/L was selected for further study. The Fe^3+^ to Fe^2+^ concentration ratios were controlled at 1:1, 1:2, 1:4, and 1:8. As depicted in Figure 2a, for the electrolyte containing Fe^3+^, the addition of Fe^2+^ caused the constant current curve to show an initial sharp increase in potential, followed by a rapid decrease. The initial stages of polarization showed a peak in the curve attributed to the oxidation of Fe^2+^ at the anode during the copper electrodeposition process, impacting the initial stability of the membrane layer. After the curve stabilized, the addition of 2 g/L Fe^2+^ resulted in the lowest stable potential. This indicates that 2 g/L Fe^2+^ further reduces the reaction potential, lowering the cell voltage during the copper electrodeposition process and thus reducing energy consumption.

As shown in Figure 2b, with the addition of Fe^2+^, the overpotential for the oxygen evolution reaction increased as the Fe^2+^oncentration increased. This indicates a decrease in the catalytic activity of the anode. Under an electrolyte condition where the Fe ion concentration was Fe^3+^:Fe^2+^ = 1:1, the oxygen evolution performance of the anode improved post-electrolysis, with a decrease in the overpotential by 24 mV compared to 2 g/L Fe^3+^. During the copper electrodeposition process, Fe^2+^ and Fe^3+^ underwent mutual conversion in the solution, with Fe^2+^ being oxidized to Fe^3+^ at the anode. The oxidation reaction at the anode caused the conversion of Fe^2+^ to Fe^3+^, and an increase in Fe^2+^ content led to a corresponding increase in Fe^3+^. Additionally, as the electrodeposition process progressed, the PbO_2_ layer on the surface gradually flaked off, along with the Fe_2_O_3_ within the PbO_2_ layer, resulting in an increase in Fe^3+^ concentration in the electrolyte. The LSV curves of the anode eventually showed the same trend as when Fe^3+^ was added alone. The cyclic voltammetry (CV) data in Figure 2c indicate that the trend of oxidation peak A, representing the oxygen evolution reaction, is consistent with the LSV results, further confirming that the addition of 2 g/L Fe^2^⁺ enhances the catalytic activity of the anode. The intensity of oxidation peak B did not show a significant decrease after the addition of 2 g/L Fe^2+^, but decreased noticeably with higher Fe^2+^ concentrations. This indicates that the presence of Fe^2+^ did not significantly impact the conversion process of PbO_x_ to PbSO_4_ within the film layer, but an increase in Fe^3+^ concentration obstructed this conversion. At a 2 g/L Fe^2+^ concentration in the electrolyte, peak C in the reduction curve showed an increase in intensity, indicating that the presence of 2 g/L Fe^2+^ enhanced the formation of PbO_2_ in the anodic film layer, thereby improving the catalytic activity of the anode. As the Fe^2+^ concentration further increased, the oxidation process at the anode led to an increase in Fe^3+^ concentration. The rise in Fe^3+^ concentration resulted in a decrease in PbO_2_ content, thereby reducing the intensity of peak C in the reduction curve.

As shown in Figure 2d,e, the self-corrosion potential increased with the addition of 2 g/L Fe^2+^. However, when the Fe^2+^ concentration was further increased to 4 g/L, the self-corrosion potential decreased sharply. The rise in PbO_2_ content also contributed to improved corrosion resistance. The composition ratio of Fe^3+^ to Fe^2+^ in the electrolyte can significantly affect the corrosion resistance of the anode. Comparing the self-corrosion potentials shown in Figure 2e, it can be observed that when the Fe^3+^:Fe^2+^ addition ratio was 1:1, the self-corrosion potential was highest. This indicates a lower thermodynamic tendency for corrosion at the anode, thereby improving its corrosion resistance. As the Fe^2+^ concentration rose, the self-corrosion potential decreased, resulting in reduced anode corrosion resistance. Figure 2f shows that the charge transfer resistance (R_ct_) decreased at 2 g/L Fe^2+^, indicating reduced hindrance to the oxygen evolution reaction of the anode and improved oxygen evolution activity. This increase in oxygen evolution activity is also attributed to the higher roughness factor (R_F_) shown in Table 2. The increased surface roughness enhanced the oxygen evolution activity of the anode. With 2 g/L Fe^2+^, the conversion of PbO_2_ on the anode accelerated, increasing the PbO_2_ content in the film layer, effectively enlarging the reaction area between the anode and the electrolyte, and enhancing the catalytic activity of the anode.

### 3.3. Anode Phase Change

**Figure 3:**
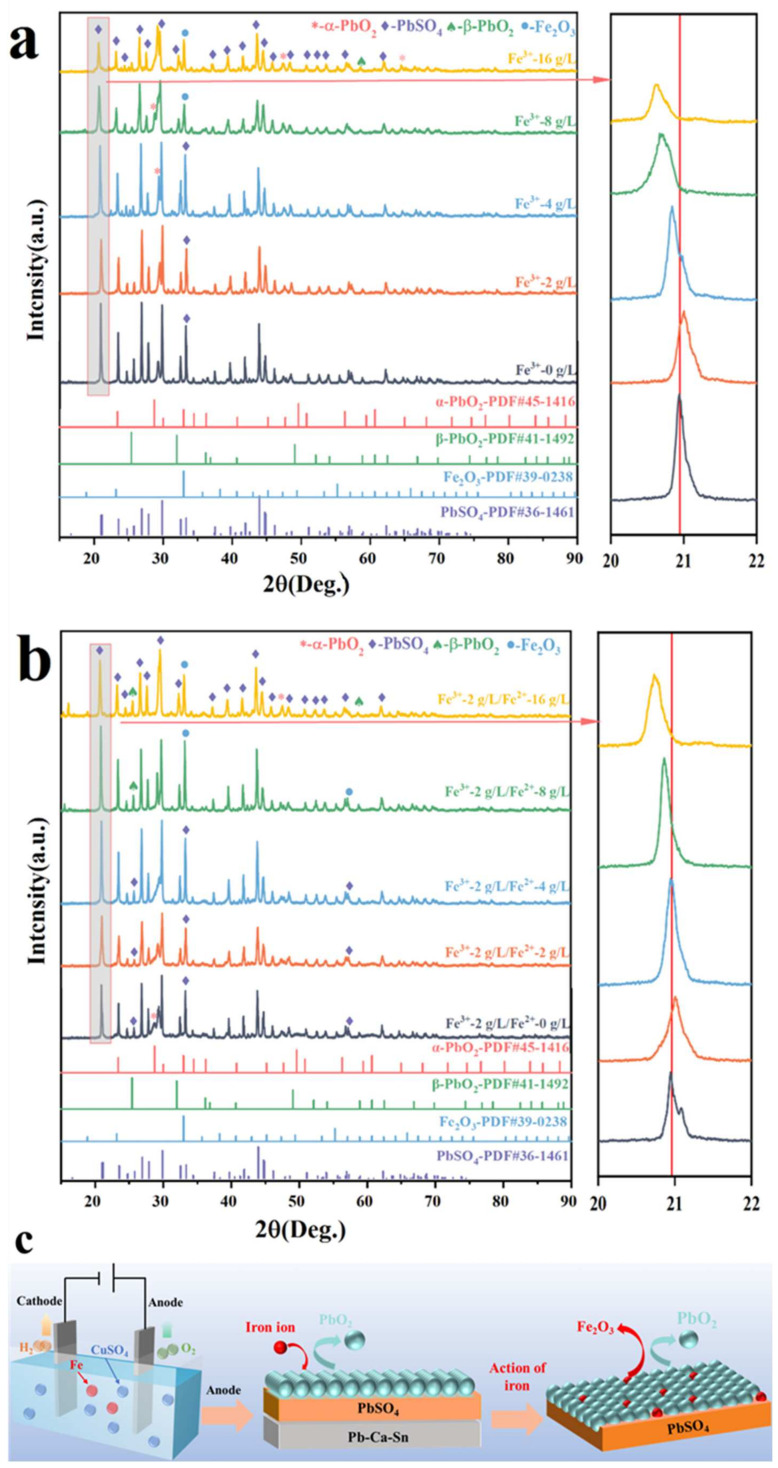
(**a**) XRD curves at different Fe^3+^ concentrations, (**b**) XRD curves at different Fe^2+^ concentrations, (**c**) anodic film layer formation process in the presence of Fe ions.

To elucidate the physical phase composition of the anode film layer and associated changes, XRD and XPS tests were conducted on anodes that underwent 48 h copper electrowinning experiments in electrolytes with varying Fe concentrations. The XRD patterns in Figure 3a,b reveal that the anode film layer primarily consisted of PbSO_4_, α-PbO_2_, and β-PbO_2_. Additionally, under high Fe ion concentrations (≥8 g/L), some Fe_2_O_3_ likely existed on the anode surface, with XRD diffraction peaks displaying rightward and leftward deviation after Fe ion addition. This suggests that Fe ions likely infiltrate the surface film layer through doping during anode film formation. At an iron ion concentration of 2 g/L, compared to when Fe³⁺ ions were not added, a rightward shift in the XRD peaks could be observed. This shift is attributed to the preferential substitution and doping of iron ions, which replace lead atoms in the lattice. Due to the larger radius of Pb atoms compared to Fe atoms, this substitution reduced the lattice spacing, resulting in the rightward shift of the diffraction peaks in the XRD pattern [25]. Consequently, the addition of Fe ions reconfigured the PbO_2_ structure on the anodic film, enhancing anode catalytic activity [26,27,28]. With increasing Fe ion concentration, a second phase, Fe_2_O_3_, eventually formed and precipitated within the film layer. The most prominent indication is the diffraction peak near 2θ = 33.3, which shifted leftward with rising Fe ion concentration and coincided with the main peak representing Fe_2_O_3_ at a 16 g/L Fe ion concentration. After the addition of Fe^3+^, XRD peak heights decreased variably, indicating that Fe^3+^ addition inhibited the crystallinity of PbSO_4_ and PbO_2_ in the surface film layers. Conversely, Fe^2+^ addition caused diffraction peaks to initially increase and then decrease, altering the crystallinity of PbSO_4_ and PbO_2_. The variation in XRD diffraction peak intensity aligns with the changes observed in the CV curves. Under the influence of Fe^3+^, the crystallinity of PbSO_4_ and PbO_2_ decreased, which impeded the transformation of PbO_x_ (0 < x < 1) to PbSO_4_ and PbO_2_. This reduction in the content of PbSO_4_ and PbO_2_ within the film layer led to a decline in the corrosion resistance of the anode. In contrast, when the concentration of Fe^2^⁺ reached 2 g/L, an increase in the crystallinity of PbSO_4_ and PbO_2_ was observed, along with an enhancement in the formation of PbO_2_, thereby improving the catalytic activity of the anode. Furthermore, the XPS spectrum in Figure S1 illustrates that the surface layer mainly comprised Pb, S, and O. However, there was a minor presence of Fe in the anode layer obtained from Fe-containing electrolytes. This also indicates that Fe ions entered the anode film layer through doping or oxide deposition.

The formation process of the anode film layer in the presence of Fe ions in the electrolyte is depicted in Figure 3c. Initially, a PbSO_4_ layer formed on the Pb-0.06%Ca-1.2%Sn anode in CuSO_4_ solution. Subsequently, PbSO_4_ continued to oxidize, transforming into a PbO_2_ film that covered the PbSO_4_ layer. Under the influence of Fe ions, some Pb atoms in the anodic PbO_2_ were replaced by Fe atoms. Concurrently, with increasing Fe^3+^ concentration, Fe_2_O_3_ became embedded in the PbO_2_ film layer on the anode, resulting in structural changes to the anode film.

### 3.4. Microscopic Morphology of Surface Film Layer

**Figure 4:**
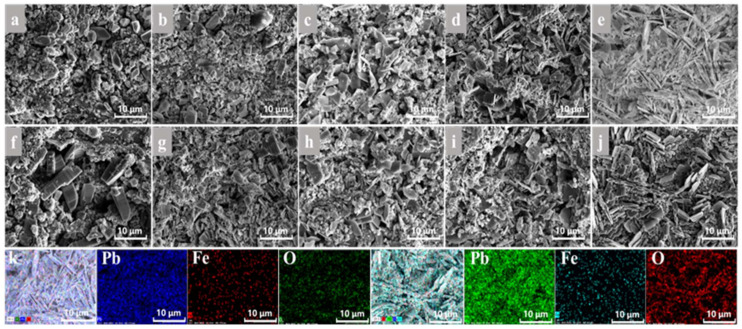
Surface morphology of membrane layer after constant current polarization (300 A/m^2^) for 48 h with different ion concentrations (**a**) Fe^3+^ 0 g/L, (**b**) Fe^3+^ 2 g/L, (**c**) Fe^3+^ 4 g/L, (**d**) Fe^3+^ 8 g/L, (**e**) Fe^3+^ 16 g/L, (**f**) Fe^3+^ 2 g/L/Fe^2+^ 0 g/L, (**g**) Fe^3+^ 2 g/L/Fe^2+^ 2 g/L, (**h**) Fe^3+^ 2 g/L/Fe^2+^ 4 g/L, (**i**) Fe^3+^ 2 g/L/Fe^2+^ 8 g/L, (**j**) Fe^3+^ 2 g/L/Fe^2+^ 16 g/L, (**k**) Fe^3+^ 16 g/L-EDS, (**l**) Fe^3+^ 2 g/L/Fe^2+^ 16 g/L-EDS.

SEM and EDS analyses were performed on the surface membrane layers of the anodes obtained from the experiment to investigate the influence of iron ions. As illustrated in Figure 4, microscopic morphology changes revealed that in the absence of Fe ions, the anode surface was enveloped by crystals with regular bulk morphology. Additionally, some foam crystals were present to enhance the density of the film layer on the anode surface, and the morphology was similar to that of the Pb-Ca-Sn alloy observed after copper electrolysis experiments [29].

Specimens subjected to experiments at varying Fe ion concentrations were analyzed using EDS (Energy Dispersive Spectroscopy). The results revealed the presence of Fe elements on the surface film layer, extensively covering the crystal surface, which is consistent with the occurrence of doping reactions. This indicates that as Fe ions were introduced, Fe ions reacted on the anode surface during electrodeposition, resulting in the substitution of Pb atoms in the PbO_2_ crystals by Fe atoms, thereby enhancing the catalytic activity of the anode. As the Fe ion concentration increased, a transition in the state of grains on the surface film layer of the lead alloy was observed, with some regular bulk structures gradually transforming into elongated, rod-shaped crystals. This phenomenon can be attributed to the inhibitory effect of Fe^3+^ on the formation of PbO_2_ in the anode film layer. The transformation in grain morphology led to a reduction in the density of the film layer, exposing an increasing area of the lead alloy substrate, which subsequently reacted continuously with the electrolyte, intensifying corrosion. This also suggests that the corrosion resistance decreased as the Fe ion concentration increased. When Fe^2+^ was added, compared to the addition of Fe^3+^ alone, the porosity of the surface film layer is reduced, the amount of PbO_2_ formed increased, and the substitution of Pb atoms by Fe led to lattice expansion in PbO_2_, improving the density of the film layer. However, as the concentration of Fe^2+^ continued to rise, the appearance of elongated, rod-shaped crystals was still observed. This was due to the increased oxidation of Fe^2+^ to Fe^3+^, which, under the influence of Fe^3+^, caused the transformation in crystal morphology, thereby reducing the corrosion resistance of the anode.

### 3.5. Potential Mechanism of Reconstruction Engineering of Pb/Fe-PbO_2_

**Figure 5:**
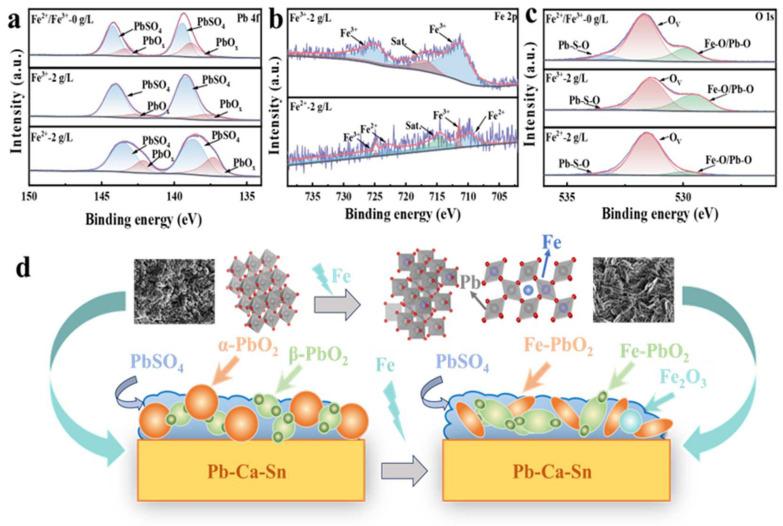
(**a**) Elemental XPS plot of Pb, (**b**) elemental XPS plot of Fe, (**c**) elemental XPS plot of O, (**d**) mechanism of anode change under the influence of Fe ions.

To analyze the chemical valence changes in each element on the anode surface, XPS analysis was conducted on anodes without Fe ions, with 2 g/L Fe^3+^, and with 2 g/L Fe^2+^. Figure 5a reveals that Pb was predominantly present as PbSO_4_ and PbO_x_. However, the addition of Fe^3+^ notably reduced the characteristic peak area representing PbO_x_, indicating a decrease in PbO_x_ content in the film layer and, subsequently, a reduction in PbO_2_ content. On the contrary, after adding 2 g/L Fe^2+^, an increase in the area of PbO_x_ was observed in the XPS spectrum of Pb. This, combined with the CV results, indicated that the presence of 2 g/L Fe^2+^ in the electrolyte increased the content of PbO_2_ to some extent. Figure 5b shows Fe predominantly appearing as Fe^3+^ in the anode surface film layer, with Fe^2+^ appearing on the anode surface upon Fe^2+^ addition [30,31]. Characteristic peaks of Fe^2+^ and Fe^3+^ appeared around 711.0 eV and 713.4 eV, respectively. With Fe^2+^ addition, the area of the XPS characteristic peaks of elemental Fe decreased, along with a decrease in Fe^3+^ content in the film layer. This indicated a reduction in Fe^3+^ incorporation into the film layer in the presence of Fe^2+^, thereby mitigating the effect of Fe^3+^ on the anode film layer. This trend is further demonstrated in Figure 5c, where the characteristic peak area located at 529 eV representing the M-O bond decreased after Fe^2+^ addition. This suggests that Fe^2+^ addition inhibited the formation of Fe-O and Pb-O bonds, reduced PbO_2_ content in the film layer, and diminished the likelihood of Fe^3+^ entering the surface film layer. Additionally, the O_v_ area increased after Fe^2+^ addition, effectively enhancing the oxygen evolution activity of the anode [32].

As depicted in Figure 5d, the presence of Fe ions in the copper electrowinning electrolyte led to the formation of a PbSO_4_/PbO_2_ structure on the anode surface. Subsequently, Fe^3+^ in the solution underwent substitutional doping after PbO_2_ formation, altering the crystal structure of PbO_2_, enhancing oxygen vacancy generation, and improving oxygen evolution activity. Anodic samples obtained after 48 h of electrolysis in a copper electrolyte solution, with a Fe^3+^ concentration of 8 g/L and under a current density of 300 A/m^2^ at 45 °C, revealed the presence of Fe_2_O_3_ phase in the surface membrane layer through XRD analysis. Additionally, the presence of Fe^3+^ in XPS confirmed that Fe ions predominantly existed in the membrane layer in the form of Fe_2_O_3_. The increase in Fe^3+^ concentration inhibited the formation of PbO_2_, leading to morphological changes in PbO_2_ on the film surface. This transformation resulted in the crystals being converted into elongated stripes that covered the PbSO_4_ layer, thereby affecting the corrosion performance of the anode and oxygen evolution reaction. The addition of 2 g/L Fe^2+^ altered the PbSO_4_/PbO_2_ content ratio in the anode layer, resulting in increased PbO_2_ content. Simultaneously, it promoted further substitutional doping of Pb atoms by Fe atoms, reduced Fe_2_O_3_ content in the film layer, and increased Fe-PbO_2_ content. This led to a higher presence of oxygen vacancies in the film layer, effectively enhancing catalytic activity.

### 3.6. Changes in Cell Voltage and Current Efficiency under the Effect of Fe Ions

**Figure 6:**
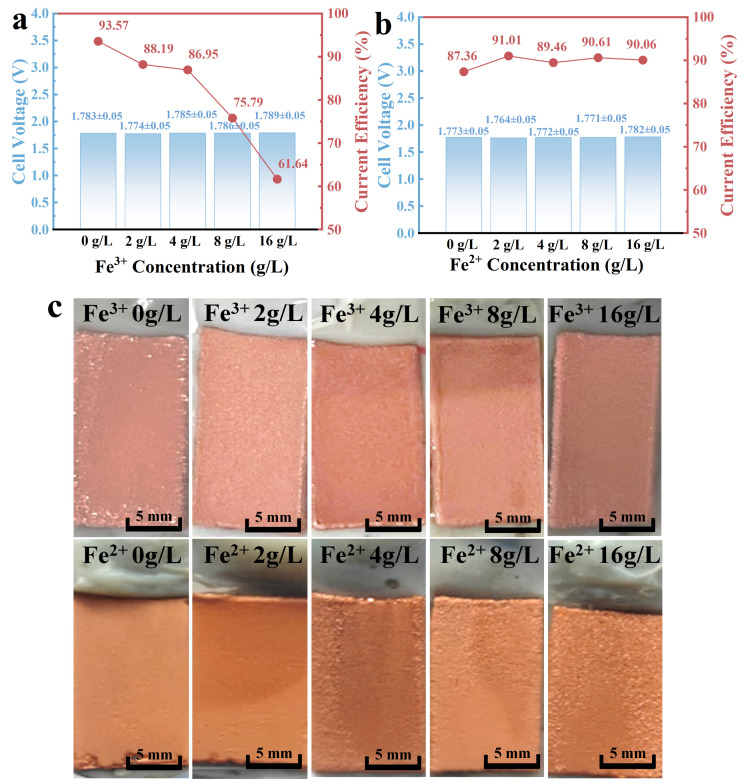
(**a**) Changes in cell voltage/current efficiency in the presence of Fe^3+^, (**b**) changes in cell voltage/current efficiency under the combined effect of Fe^3+^ and Fe^2+^, (**c**) copper cathodes with different Fe ion concentration electrolyte.

A 48 h copper electrodeposition experiment was conducted to further investigate the impact of iron ions on cell voltage and current efficiency during the production process, and the obtained cell voltage and current efficiency are shown in Figure 6a,b.

After adding Fe^3+^, the current efficiency decreased with the increase in Fe^3+^ concentration. The addition of Fe^2+^ induced an increase in current efficiency, which was highest at a Fe^2+^ concentration of 2 g/L. The current efficiency of the electrowinning cell was higher than that of the electrowinning cell with the addition of Fe^3+^. The change in current efficiency can be attributed to the change in charge transfer resistance R_ct_, and an increase in R_ct_ led to a decrease in current efficiency. The cell voltages were averaged over several tests, and the results show that the cell voltage was relatively lowest after adding 2 g/L of Fe^3+^ and Fe^2+^. The anode at this concentration had the best oxygen evolution activity, which reduced energy consumption and cost in the copper electrowinning process. As shown in Figure 6c, the addition of Fe ions favored the smoothing of the cathode copper surface to obtain smoother cathode copper, where the smoothest cathode copper was obtained by adding both 2 g/L Fe^3+^ and Fe^2+^.

## 4. Conclusions

The influence of Fe ions in the copper electrowinning process significantly impacted the electric chemical behavior of the anode. This study examined the effects of varying Fe ions (Fe^2+^ and/or Fe^3+)^ concentrations on conventional Pb−Ca−Sn anodes. Copper electrodeposition experiments were conducted for 48 h under a current density of 300 A/m^2^ at 45 °C in copper electrolyte solutions with varying Fe^3+^ concentrations. The aim was to ensure the formation of stable membrane layers on Pb−Ca−Sn anodes. The study focused on investigating the electrochemical behavior and structural changes of these membrane layers. The results are summarized as follows:

(1) The addition of Fe^3+^ inhibits the formation of PbO_2_ and PbSO_4_ in the film layer, and excessively high concentrations of Fe^3+^ lead to a reduction in the corrosion resistance of anode and catalytic activity. When the Fe^2+^ concentration is controlled around 2 g/L, the oxygen evolution catalytic activity of the anodic film layer is enhanced.
(2) When 2 g/L of Fe^2+^ is present in the electrolyte, the PbO_2_ content in the anode film layer increases, improving both the catalytic activity and corrosion resistance of the anode. However, as the Fe^2+^ concentration increases, the Fe^3+^ concentration in the electrolyte also rises, leading to a decline in the catalytic activity of anode and corrosion resistance.
(3) When the concentration of iron ions (Fe^3+^ and Fe^2+^) is controlled at 2 g/L, the catalytic activity of the anode is enhanced. This enhanced catalytic activity is attributed to the doping of iron ions. Substitutional doping induces changes in the PbO_2_ crystal structure and increases the content of oxygen vacancies in the film, thereby improving the catalytic activity of the anode.

## Figures and Tables

**Table 1:** Parameters associated with the EIS fitting mapping.

Fe^3+^ Concentration (g/L)	R_s_ Ω·cm^2^	R_ct_ Ω·cm^2^	Q_dl_ Ω^−1^·cm^−2^·s^n^	C_dl_ μF·cm^−2^	n	R_F_
0	0.70	13.75	149,150	3278.928	0.67	163.9
2	0.89	17.93	104,500	11,314.369	0.80	565.7
4	1.60	18.63	63,221	9951.288	0.84	497.1
8	1.73	33.11	69,344	9959.024	0.80	497.9
16	1.51	144.8	33,972	3161.026	0.78	158.1

**Table 2:** Parameters associated with the EIS fitting mapping.

Fe^2+^ Concentration (g/L)	R_s_ Ω·cm^2^	R_ct_ Ω·cm^2^	Q_dl_ Ω^−1^·cm^−2^·s^n^	C_dl_ μF·cm^−2^	n	R_F_
0	2.20	36.03	65,204	8820.071	0.80	441.0
2	3.87	29.76	35,765	10,368.597	0.89	518.4
4	2.33	53.07	60,587	8001.658	0.83	400.1
8	2.58	95.2	54,936	7548.613	0.83	377.4
16	5.59	125.6	31,470	6243.303	0.87	312.2

## Data Availability

The original contributions presented in the study are included in the article/Supplementary Materials, further inquiries can be directed to the corresponding authors.

## Supplementary Materials

The following supporting information can be downloaded at: https://www.mdpi.com/article/10.3390/molecules29194578/s1, Figure S1: XPS gross spectra at different Fe ions concentrations.
