# Supplementary material for: Influence of Fe Ions on Anode Performance and the Mechanism of Action during Copper Electrowinning Process
Source: Molecules. 2024 Sep 26;29(19):4578. doi: 10.3390/molecules29194578 (PMC11478191; doi:10.3390/molecules29194578)
Supplement: Supplementary file 1 [file molecules-29-04578-s001.zip › molecules-3203374-supplementary.pdf]

Supplementary Material

Influence of Fe Ions on Anode Performance and the Mechanism of Action during Copper Electrowinning Process

Cheng Jiang[1,4], Yiwen Chen [1,2], Yingping Zhou[2,3], Buming Chen[1,2,3*], Hui Huang[1,2,3], Jun Guo[1,2,3*], Chao Gao[1,2,3], Ruidong Xu[1,2], Zhongcheng Guo[1,2,3]

1 Faculty of Metallurgical and Energy Engineering, Kunming University of Science and Technology, Kunming 650093, China

2 Research Center of Metallurgical Electrode Materials Engineering Technology, Yunnan Province, Kunming 650106, China

3 Kunming Hendera Science and Technology Co., Ltd., Kunming, 650106, China

4 State Key Laboratory of Comprehensive Utilization of Low-Grade Refractory Gold Ores, Xiamen, 361101, China

***Corresponding Author: bumchen@kust.edu.cn (B. Chen); 1038gj@kust.edu.cn (J. Guo);**

The supporting information includes 2 pages, 1 figure.

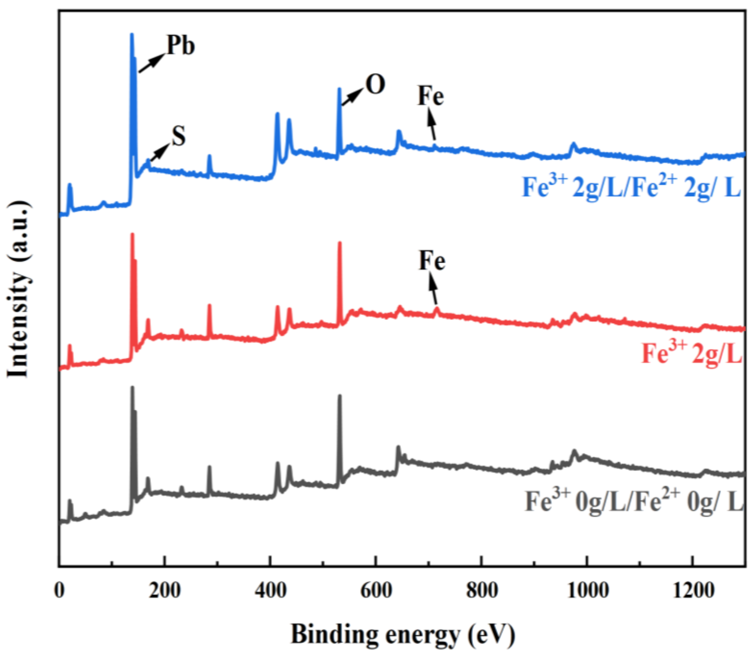

Figure S1. XPS gross spectra at different Fe ions concentrations
